# microRNA-32 induces radioresistance by targeting DAB2IP and regulating autophagy in prostate cancer cells

**DOI:** 10.3892/ol.2015.3551

**Published:** 2015-07-30

**Authors:** HAIQIU LIAO, YANG XIAO, YINGBIN HU, YANGMING XIAO, ZHAOFA YIN, LIANG LIU

**Affiliations:** 1 Department of Urology, Loudi Central Hospital of Hunan, Loudi, Hunan 417000, P.R. China; 2 Department of Orthopaedics, Loudi Central Hospital of Hunan, Loudi, Hunan 417000, P.R. China; 3 Department of Colorectal Surgery, The Affiliated Cancer Hospital of Xiangya School of Medicine, Central South University, Changsha, Hunan 410013, P.R. China; 4 Department of Oncology, Loudi Central Hospital of Hunan, Loudi, Hunan 417000, P.R. China

**Keywords:** microRNA-32, DAB2IP, autophagy, apoptosis, radioresistance, prostate cancer

## Abstract

The aberrant expression of microRNAs (miRNAs/miRs) has been found in numerous cancer types. miR-32 is an oncomiR in prostate cancer (PCa), however, the mechanisms by which miR-32 functions as a regulator of radiotherapy response and resistance in PCa are largely unknown. In the present study, it was found that DAB2 interacting protein (DAB2IP), the miR-32-dependent tumor-suppressor gene, was downregulated and induced autophagy and inhibited radiotherapy-induced apoptosis in PCa cells. miR-32 expression was upregulated or overexpressed in PCa, and miR-32 inhibited DAB2IP expression through a direct binding site within the DAB2IP 3′ untranslated region. miR-32 mimics enhanced tumor cell survival and decreased radiosensitivity in the PCa cells, which were reversed by miR-32 inhibitor. Flow cytometric analysis revealed that overexpressed miR-32, consistent with the DAB2IP-knockdown results, reduced ionizing radiation (IR)-induced cell apoptosis, which was restored by 4 nM brefeldin A treatment. More significantly, the overexpression of miR-32 and the knockdown of DAB2IP enhanced autophagy in the IR-treated PCa cells. miR-32 regulated the expression of autophagy-related proteins, such as DAB2IP, Beclin 1 and Light chain 3β I/II, as well as phosphorylation of S6 kinase and mammalian target of rapamycin. In conclusion, these data provide novel insights into the mechanisms governing the regulation of DAB2IP expression by miR-32 and their possible contribution to autophagy and radioresistance in PCa.

## Introduction

Prostate cancer (PCa) is the most frequently diagnosed cancer and the second leading cause of cancer-associated mortality among men in the United States (1). Current medical management for localized PCa ranges from close monitoring for indolent disease to radical treatments, such as radiation therapy (RT) or surgery. RT provides excellent local control and increased overall survival rates for PCa. However, a significant number of high-risk patients will fail therapy, develop resistance and eventually succumb to the disease (2,3). With an increased level of knowledge with regard to biomarkers and their effect on therapeutic response, in the future, physicians may be able to personalize care based on a patient's biomarkers.

DAB2 interacting protein (DAB2IP), also known as aspartokinase (ASK1)-interacting protein-1, is a novel member of the Ras GTPase-activating protein family and is downregulated, with growth inhibitory and apoptosis enhancing activities, in PCa (4). Downregulation of DAB2IP, mainly due to epigenetic regulation, inversely correlates with tumor grade and predicts PCa progression (5,6). DAB2IP is a unique scaffold protein that modulates a variety of biological activities, including cell growth, apoptosis and survival via the phosphoinositide 3-kinase-Akt, Wnt-epithelial-mesenchymal transition, ASK-JNK, Ras-mitogen-activated protein kinase, and nuclear factor-κB pathways in PCa (4,7–9). By knocking down endogenous DAB2IP levels, PCa cells could gain proliferative potential and become resistant to stress-induced apoptosis (6). An increasing number of studies have shown that DAB2IP plays an important role in the radioresistance and chemoresistance of PCa (1,10). DAB2IP regulates Clusterin gene expression via cross-talk between Wnt/β-catenin and insulin-like growth factor-I (IGF-I)/IGF receptor signaling in metastatic castration-resistant PCa (10). DAB2IP loss has been shown to result in resistance to ionizing radiation (IR) due to enhanced DSB repair and apoptosis resistance (1,11). Recently, a novel function of DAB2IP was shown in suppressing IR-induced and DNA-PKcs-associated autophagy, and promoting apoptosis in PCa cells (12). However, the regulatory mechanism of DAB2IP on the radioresistance in PCa has not been well clariﬁed. Thus, an increased level of knowledge with regard to the molecular mechanisms of DAB2IP in PCa therapy resistance could aid in the identification of significant novel therapeutic targets for advanced disease.

microRNAs (miRNAs/miRs), an abundant class of ~22-nucleotide small non-coding RNAs, post-transcriptionally regulate gene expression through binding to multiple target mRNAs (13–15). Extensive PCa miRNA profiling has shown that a number of miRNAs are differentially expressed between PCa and adjacent normal tissues, thus contributing to PCa progression (16,17). Therefore, understanding the molecular mechanisms by which these miRNAs act in the deregulation of cellular signaling in PCa cells may assist in the development of improved therapeutic strategies disease treatment. To date, however, the mechanism behind the involvement of the miRNA-dependent DAB2IP pathway in the radioresistance of PCa has not been investigated.

In the present study, the regulatory effect of miR-32 on PCa cell survival and apoptosis was determined during radiotherapy, and the involved pathways were analyzed.

## Materials and methods

### 

#### Cells and specimens

PCa cell lines (DU145 and PC3) and a normal prostate cell line (RWPE-1) were grown in T medium (Invitrogen Life Technologies, Carlsbad, CA, USA) with 5% fetal bovine serum (HyClone, Hudson, NH, USA) at 37°C in a 5% CO_2_ humidified chamber.

Human PCa tissues, adjacent non-tumor tissues (located 2.5 cm from the tumor) and normal PCa tissues were obtained from patients diagnosed with PCa in Department of General Surgery, Loudi Central Hospital of Hunan (Loudi, China). The specimens were obtained after surgical resection and immediately frozen at −80°C until use. The study methodologies conformed to the standards set by the Declaration of Helsinki. Collection and usage of all specimens were approved by the Loudi Central Hospital Ethics Committee (Loudi, China).

#### miRNAs and transfection

The *Homo sapien* (has)-miR-32 mimics, has-miR-32 inhibitor and has-miR-32 scrambled negative control were obtained from GeneCopoeia, Inc. (Rockville, MD, USA). miR-32 mimics and miR-32 inhibitor were used to increase and decrease the expression of miR-32, respectively. miRNA transfection was performed using Lipofectamine 2000 transfection reagent (11668-019; Invitrogen Life Technologies). In brief, cells were plated in a 24-well plate and incubated overnight to achieve 80–90% confluence at the time of transfection. In each well, 5 µl miRNA was added to 50 µl Opti-MEM® (31985070; Gibco Life Technologies, Carlsbad, CA, USA). Separately, 2 µl Lipofectamine 2000 transfection reagent was added to 50 µl Opti-MEM and mixed gently. The transfection complex was added to the cells and incubated for 6 h at 37°C in a 5% CO_2_ incubator, after which the serum-containing medium was replaced.

To confirm the effect of the miRNAs on the expression of miR-32, reverse transcription-quantitative polymerase chain reaction (RT-qPCR) was performed to determine the mRNA expression levels of miR-32 in the PCa cell lines. The RT-qPCR primers (hsa-miR-32-5p; cat. no. HmiRQP0404) were obtained from GeneCopoeia, Inc. and the cycling conditions were as follows: Step 1, 95°C for 30 min; and step 2, 40 cycles of 95°C for 15 sec then 58°C for 35 sec. The mRNA copy number results obtained were recalculated per 1 µg total RNA. The transfected cells were the expanded and harvested for the following further analyses.

#### IR treatment

All cells were irradiated in ambient air using a cesium-137 source (Mark 1–68 irradiator; J.L. Shepherd and Associates, San Fernando, CA, USA) at 2 Gy at room temperature for 24 h.

#### Luciferase reporter assay

The cells were lysed in passive lysing buffer and then analyzed for firefly and Renilla luciferase activities using the commercial Dual-Luciferase Reporter Assay System (E1910; Promega Corporation, Madison, WI, USA) according to the manufacturer's instructions. Firefly luciferase activity was normalized to the Renilla luciferase activity.

#### Western blot analysis

Whole cell extracts were prepared with a cell lysis reagent (Sigma-Aldrich, St. Louis, MO, USA) according to the manufacturer's instructions, and then the protein was quantified by a bicinchoninic acid assay (Pierce, Rockford, IL, USA). Next, the protein samples were separated by 10% SDS-PAGE and detected using western blot analysis. Mammalian target of rabbit anti-human polycolonal rapamycin (mTOR), phosphor-mTOR (pmTOR, S2448), phospho-S6 kinase (pS6K, T389), Light chain 3β (LC3B), Beclin 1 and DAB2IP antibodies were purchased from Cell Signaling Technology Inc. (1:1,000 dilution; Danvers, MA, USA). Mouse anti-actin monoclonal antibody was purchased from Sigma-Aldrich (1:2,000 dilution). Fluorescent dye-conjugated secondary antibodies were obtained from Invitrogen Life Technologies.

#### RNA isolation and RT-qPCR

Total RNA was extracted using TRIzol (15596-026; Invitrogen Life Technologies), according to the manufacturer's instructions, and then reverse transcribed for quantification using the TaqMan microRNA Reverse Transcription kit (4366596; Applied Biosystems Life Technologies, Foster City, CA, USA) according to the manufacturer's instructions. Mature miRNAs were quantified using 2-step TaqMan RT-qPCR with the TaqMan microRNA kit. The miRNA expression level was normalized using U6 small nuclear RNA (HmiRQP9001) as an internal control, as previously described (18).

#### Cell survival and apoptosis assay

The cells were diluted serially to 5.0×10^5^ cells per 60 mm-diameter well and plated into dishes (area, 2,827.43 mm^2^) in triplicate. Cells were pretreated with brefeldin A (BFA) to a final concentration of 4 nM. After 3 h of incubation at 37°C in a 5% CO_2_ atmosphere, the cells were treated with increasing doses of IR, to a total dose of 2 Gy. After 0 to 48 h, cell viability was estimated by MTT assay (19). Cell apoptosis was assessed using the ANXA5-FITC Apoptosis Detection kit (556570; BD Pharmingen, San Diego, CA, USA) by flow cytometric analysis, as previously described (4).

#### Statistical analysis

Each experiment was repeated at least three times. Data are shown as the mean ± standard deviation, and were analyzed using SPSS 18.0 (SPSS, Inc., Chicago, IL, USA). Statistical comparisons between groups were analyzed using the *t*-test and two-tailed P<0.05 was considered to indicate a statistically signiﬁcant difference.

## Results

### 

#### Increased expression of miR-32 in PCa

**Figure 1.**
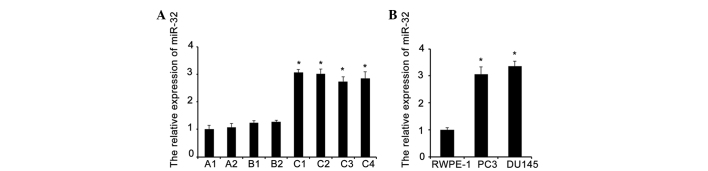
Expression of miR-32 is upregulated in prostate cancer (PCa). (A) The expression level of miR-32 in normal tissues (A1 and A2), matched adjacent non-tumor tissues (B1 and B2) and PCa tissues (C1, C2, C3 and C4) were detected by reverse transcription-quantitative polymerase chain reaction. U6 small nuclear RNA was used as an endogenous control. The miR-32 levels were significantly upregulated in the PCa tissues. (B) The relative expression level of miR-32 in the normal prostate cell line and two PCa cell lines. miR, microRNA.

As the expression of miR-32 was reported to increase significantly in PCa tissue in a previous study (16), the present study examined the expression of miR-32 in normal prostate tissues, human PCa tissues and adjacent non-tumor tissues, as well as two PCa cell lines (PC3 and DU145) and a normal prostate cell line (RWPE-1), using RT-qPCR. miR-32 expression was frequently upregulated or overexpressed in the PCa tissues and cell lines compared with the normal tissues and RWPE-1 cell line (P=0.004; Fig. 1).

#### DAB2IP is a target of miR-32 in PCa cells

**Figure 2.**
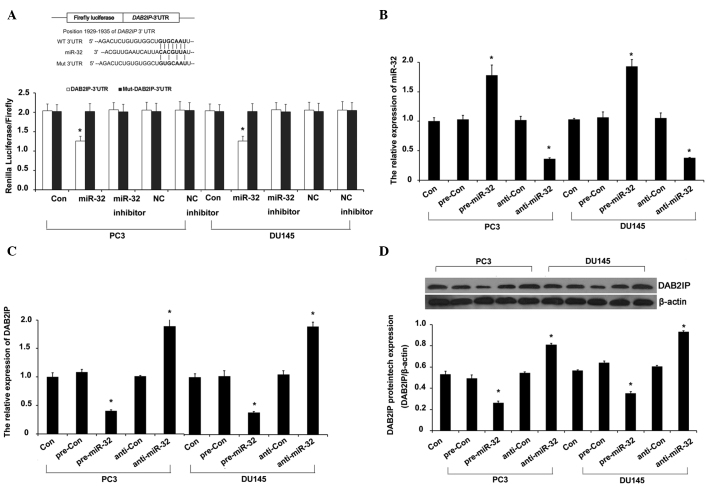
miR-32 targets DAB2IP. (A) Schema representing the functional interaction between miR-32 and the seed sequence (bold) in the 3′-UTR of DAB2IP, as predicted by TargetScan. Luciferase assay of PC3 and DU145 cotransfected with reporter constructs containing DAB2IP 3′-UTRs with (DAB2IP-3′-UTR) or without (Mut-DAB2IP-3′-UTR) miR-32 binding sites and miR-32 mimic, miR-32 inhibitor or scrambled control miRNA for 72 h. (B and C) The level of miR-32 was assayed by TaqMan reverse transcription-quantitative polymerase chain reaction (RT-qPCR). In parallel, the mRNA level of DAB2IP was assayed by qRT-PCR. (D) The level of DAB2IP protein expression was assayed by western blotting. PC3 and DU145 cells were transfected with inhibitor or mimic of miR-32 or inhibitor control or mimic control (100 nM) for 72 h. Error bars represent the standard deviation from the mean. *P<0.01 vs. control. miRNA, microRNA; DAB2IP, DAB2 interacting protein; NC, negative control of miR-32 group; NC inhibitor, negative control of miR-32 inhibitor group; UTR, untranslated region; con, control; pre-miR-32, miR-32 mimic; pre-con, mimic control; anti-miR-32, miR-32 inhibitor; anti-con, inhibitor control.

Previous studies indicated that DOC-2/DAB2IP is important in the development of radioresistance in PCa through the induction of autophagy (10,12). To investigate the diverse and complex network of upstream signaling pathways of DAB2IP-mediated autophagy in radioresistance in PCa, the present study screened the candidate miRNAs that may target DAB2IP using TargetScan (www.targetscan.org) and miRanda (www.microrna.org) software. miR-32 was shown to pair well with and target the 3′ untranslated region (3′-UTR) of DAB2IP, with strong evidence provided by the reporter assay and RT-qPCR of the present study (P<0.009; Fig. 2A). To further analyze the targeted regulation of miR-32 on DAB2IP in the IR-treated PCa cells, the miR-32 level was manipulated by miR-32 mimic and inhibitor transfection. The miR-32 level was significantly elevated in the miR-32 mimic group, but significantly decreased in the miR-32 inhibitor group of the PCa PC3 and DU145 cell lines (P=0.007; Fig. 2B). The miR-32 mimic transfection significantly reduced the DAB2IP expression at the mRNA (Fig. 2C) and protein (Fig. 2D) levels in the PC3 and DU145 cells (P=0.007). These results confirmed that miR-32 regulated DAB2IP by targeting its 3′-UTR and suppressing its translation.

#### miR-32 contributes to the radioresistance of PCa cells

**Figure 3.**
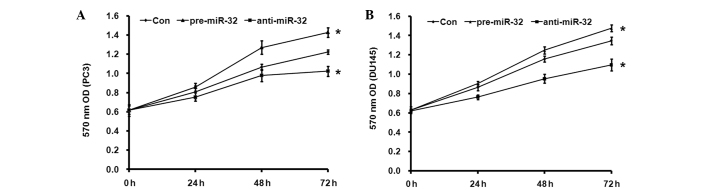
miR-32 decreases the radiosensitivity in PC3 and DU141 cell lines. MTT analysis of (A) PC3 and (B) DU145 cells following combined treatment with miR-32 mimic/inhibitor and IR as indicated. Error bars represent the standard deviation from the mean. *P<0.01 vs. control. miR, microRNA; OD, optical density. con, control; pre-miR-32, miR-32 mimic; anti-miR-32, miR-32 inhibitor.

To test the effect of miR-32 on the radiosensitivity of PCa cells, PC3 and DU145 cells were employed. As shown in Fig. 3, the silencing of endogenous miR-32 resulted in a significant radiation-sensitizing effect in the PC3 and DU145 cells (P=0.008). By contrast, the overexpression of miR-32 increased the radiation resistance of the PC3 and DU145 cells (P=0.008; Fig. 3). These *in vitro* results indicated that miR-32 can increase the resistance of PC3 and DU145 cells to radiation.

#### miR-32 inhibits apoptosis by modifying DAB2IP expression

**Figure 4.**
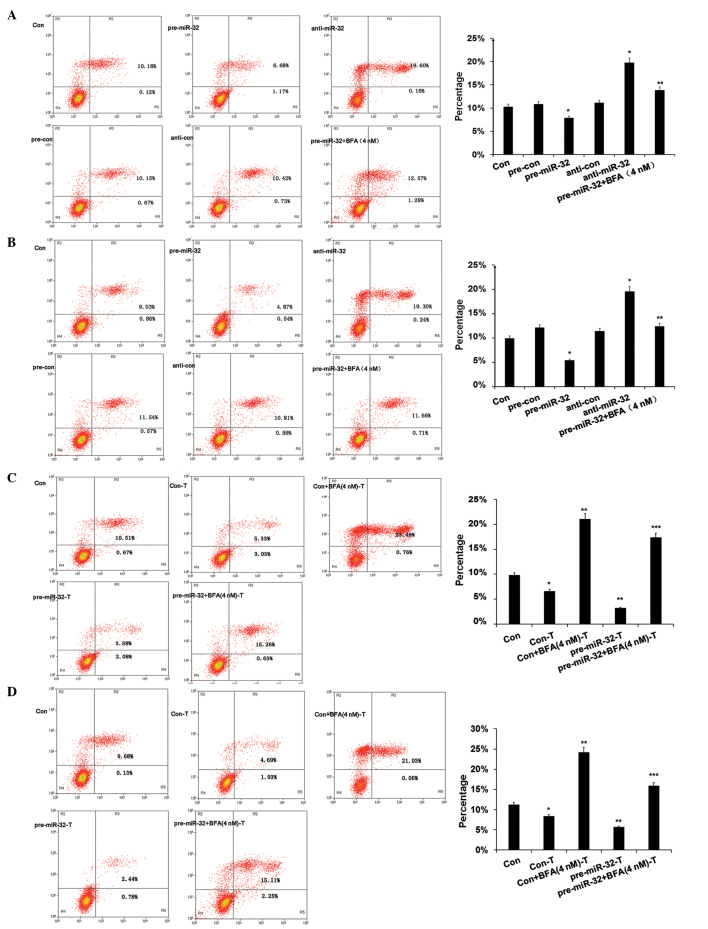
miR-32 reduces apoptosis through suppression of DAB2IP expression. Apoptosis was analyzed by flow cytometry. (A) PC3 and (B) DU145 cells were transfected with miR-32 mimic (pre-miR-32), mimic control (pre-con), miR-32 inhibitor (anti-miR-32) and inhibitor control (anti-con). *P<0.01 vs. control; **P<0.01 vs. pre-miR-32 group. (C) PC3 and (D) DU145 cells were treated with TALEN (T) knockout of DAB2IP. *P<0.01 vs. control; **P<0.01 vs. Con-T group; ***P<0.01 vs. pre-miR-32-T group and Con+BFA (4 nM)-T group. Error bars represent the standard deviation from the mean. miR, microRNA; DAB2IP, DAB2 interacting protein; BFA, brefeldin A.

In previous studies, DAB2IP-knockdown cells were resistant to radiation-induced apoptosis in PCa (1,12). The present study investigated whether miR-32 mimic treatment and the suppression of DAB2IP expression by endogenous DAB2IP knocked down the resistance of PCa to radiotherapy. PCa cells were treated with 2 Gy IR for 24 h. As shown in Fig. 4A, cell apoptosis was significantly upregulated in the miR-32 deficient group (anti-miR-32) and downregulated in the miR-32 overexpression group (pre-miR-32; P=0.006). The PCa cells were further subjected to IR combined with an apoptosis promoter, BFA. Flow cytometry assay clearly showed that BFA treatment restored radiation-induced cell death when miR-32-overexpressing PCa cells and BFA-treated miR-32-overexpressing PCa cells were compared (P=0.004). Similar to miR-32 overexpression, DAB2IP-knockdown treatment reduced apoptosis (P=0.009; Fig. 4C). BFA (4 nM) could restore cell apoptosis in the DAB2IP-deficient cells. On the basis of these results, we propose that the increased resistance to IR in miR-32-overexpressing and DAB2IP-knockdown PCa cells may be partially due to the inhibition of apoptosis.

#### miR-32 induces autophagy via the mTOR-S6K pathway

**Figure 5.**
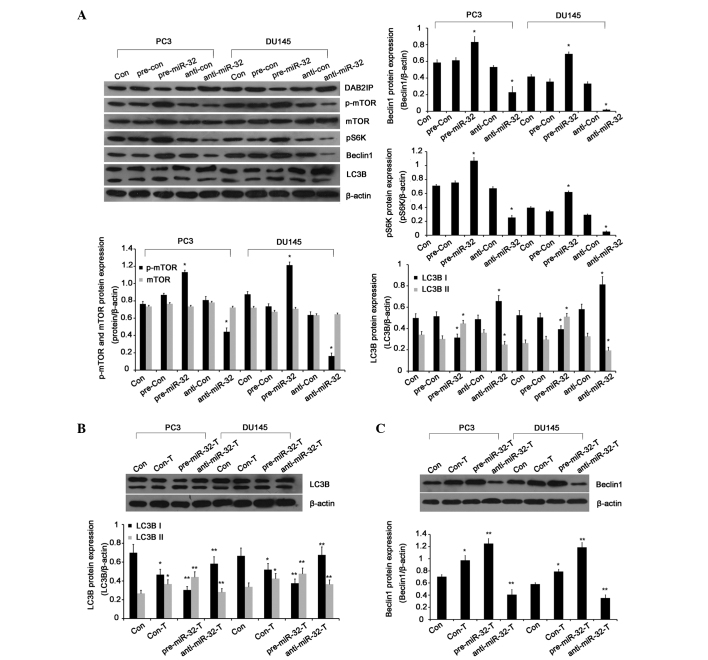
miR-32 induces autophagy via the mTOR-S6K Pathway. (A) The phosphorylation of mTOR and S6K and the expression of autophagy-associated Beclin 1 and LC3B were determined by Western blot analysis at 24 h post-IR (2 Gy). *P<0.01 vs. control. DAB2IP regulates autophagy-associated proteins (B) LC3B and (C) Beclin 1. *P<0.01 vs. control. Error bars represent the standard deviation from the mean. **P<0.01 vs. Con-T group. miR, microRNA, p-, phosphorylated; mTOR, mammalian target of rapamycin; S6K, S6 kinase; LC3B, light chain 3β; T, TALEN; pre-miR-32, miR-32 mimic; pre-con, mimic control; anti-miR-32, miR-32 inhibitor; anti-con, inhibitor control.

Given that DAB2IP acts as an autophagy inhibitor in radioresistant PCa cells, the present study investigated the function of miR-32 as a regulator of PCa autophagy. Beclin1 and LC3B are autophagy-related markers and are critical for regulating autophagy. LC3B exists in a cytosolic form, LC3B-I, and a form that is conjugated to phosphatidylethanolamine, LC3B-II (20,21). Increased LC3B-II levels are closely associated with the number of autophagosomes and serve as a good indicator of autophagosome formation (22). To investigate the role of miR-32 in autophagy, the expression levels of the microtubule-associated proteins Beclin1 and LC3B were determined. As shown in Fig. 5A, silenced miR-32 impaired the IR-mediated induction of LC3B-II in the PCa cells. In addition, DAB2IP-knockdown decreased the expression of LC3B-I and increased the expression of LC3B-II, which were revised by miR-32 inhibitor (P=0.004 and P=0.007, respectively; Fig. 5B). Moreover, the higher expression of Beclin 1 protein was noted in the miR-32 overexpressing and DAB2IP-knockdown PCa cells (P=0.006 and P=0.005, respectively; Fig. 5C).

It has been reported that the Akt-mTOR pathway negatively regulates autophagy (23,24). To investigate how miR-32 regulated IR-induced autophagy in the PCa cells, the phosphorylation of mTOR was measured in the two cell lines. Fig. 5A shows the marked inhibition of phosphorylated mTOR in miR-32-silenced cells (P=0.007). Furthermore, studies have shown that S6K is a critical downstream effector of the mTOR signaling pathway (25). In the present study, increased phosphorylation of S6K was observed in the miR-32-overexpressing PCa cells and decreased phosphorylation of S6K was observed in the miR-32-silenced PCa cells (P=0.006; Fig. 5A). Although mTOR-S6K activation is known to suppress autophagy in mammalian cells, emerging studies have indicated that, in certain situations, the mTOR-S6K pathway positively regulates autophagy (26–28). Together, the findings indicate that the miR-32 may promote IR-induced autophagy through the mTOR-S6K pathway.

## Discussion

PCa, the second most commonly occurring cause of cancer-related mortality among men in the majority of countries, is a complex and multifactorial disease (29). Current medical management for localized PCa ranges from close monitoring for indolent disease to treatments such as radiotherapy, chemotherapy or surgery. Treatment with radiation therapy has the advantages of being non-invasive and well tolerated. However, radiotherapy resistance is also a common occurrence and contributes to the failure in blocking disease progression (30). Accumulating studies have evaluated the resistance mechanisms and biological factors that are involved (31,32). Autophagy is an intracellular self-protective mechanism that functions by preventing the toxic accumulation of damaged components and by recycling these components to sustain metabolic homoeostasis (33,34). Upregulated autophagy has been identified in a wide variety of cancer cells that undergo metabolic and therapeutic stress, and the process contributes to the resistance to chemotherapy by a range of tumor types (35,36). The blocking of autophagy in cancer cells is emerging as a novel approach to enhance the sensitivity of therapy in cancers (37). A previous study confirmed the important role of DAB2IP in the autophagy inhibition of PCa cells with IR-treatment *in vitro* (12). DAB2IP, a potential tumor suppressor gene, is often downregulated in PCa primarily due to altered epigenetic regulation of its promoter (38). Recent studies have indicated that the loss of DAB2IP expression in PCa cells greatly increases radiation resistance *in vitro* (1), and the overexpression of DAB2IP suppresses IR-induced autophagy and promotes apoptosis in PCa cells (12). However, the regulation of DAB2IP, particularly by miRNAs, remains largely unknown. miRNAs have been detected in association with cancer diagnosis and prognosis as promising biomarkers and regulators of tumor proliferation, invasion, migration and apoptosis. miR-218, as a tumor-suppresser, inhibits cancer cell migration and invasion via the targeting of LASP1 in PCa (39). miR-20a promotes the invasion and migration of PCa via the targeting of ABL2 (40). miR-494-3p targets CXCR4 in order to the proliferation, invasion and migration of PCa (41). miR-124 exhibits anti-proliferative and anti-aggressive effects on PCa cells via the PACE4 pathway (42). These miRNAs often function as oncogenes by repressing tumor suppressors or function as suppressors by negatively regulating oncogenes, indicating the potential effects on the prognosis and clinical application to PCa therapy. The present study established for the ﬁrst time the important role played by miR-32 in inhibiting the expression of the DAB2IP tumor suppressors in PCa. It was demonstrated that miR-32 was well paired with the 3′-UTR of DAB2IP. Functional analysis demonstrated that PCa cells post-DAB2IP blockage by miR-32 were more resistant to IR treatment, with increased cell proliferation and reduced cell apoptosis.

A previous study showed that DAB2IP mediated the radiosensitization of PCa cells partially through the inhibition of autophagy (12). DAB2IP was involved in the autophagy pathway and overexpression of this gene attenuated IR-induced autophagy (12). In the present study, it was observed that LC3B and Beclin 1 were upregulated in the PCa cells with silenced DAB2IP and overexpressed miR-32. The mTOR-S6K pathway is postulated to be a negative regulator of mammalian autophagy (19). The mTOR-S6K pathway was recorded as inactivated in PCa cells with downregulated DAB2IP expression (12). Activated mTOR induced mTOR complex 1 substrate S6K phosphorylation, which resulted in the induction of the functional protein translational machinery (43). Consistent with this study, the present results showed that the phosphorylation of S6K was increased in the miR-32-overexpressed PCa cells. Therefore, we speculate that miR-32 enhanced the radioresistance of the PCa cells by promoting DAB2IP-related autophagy via the mTOR-S6K pathway.

In conclusion, the present study demonstrated that miR-32 directly targeted DAB2IP in PCa, and induced DAB2IP-deficient radioresistant human PCa cells. Moreover, the findings demonstrated the critical role of miR-32 in inhibiting the mTOR-S6K pathway and suppressing autophagy by targeting DAB2IP. On the basis of these results, miR-32 appears to be a novel tumor promoter and plays an important role in radiotherapy resistance during the treatment of PCa.

## References

[1] Kong Z, Xie D, Boike T, Raghavan P, Burma S, Chen DJ, Habib AA, Chakraborty A, Hsieh JT, Saha D (2010). Downregulation of human DAB2IP gene expression in prostate cancer cells results in resistance to ionizing radiation. Cancer Res.

[2] Hanks GE, Pajak TF, Porter A (2003). Phase III trial of long-term adjuvant androgen deprivation after neoadjuvant hormonal cytoreduction and radiotherapy in locally advanced carcinoma of the prostate: The radiation therapy oncology group protocol 92–02. J Clin Oncol.

[3] Roach M (2005). Radiotherapy plus adjuvant goserelin improves survival in men with poor prognosis prostate cancer. Cancer Treat Rev.

[4] Xie D, Gore C, Zhou J, Pong RC, Zhang H, Yu L, Vessella RL, Min W, Hsieh JT (2009). DAB2IP coordinates both PI3K-Akt and ASK1 pathways for cell survival and apoptosis. Proc Natl Acad Sci USA.

[5] Chen H, Toyooka S, Gazdar AF, Hsieh JT (2003). Epigenetic regulation of a novel tumor suppressor gene (hDAB2IP) in prostate cancer cell lines. J Biol Chem.

[6] Wu K, Liu J, Tseng SF (2014). The role of DAB2IP in androgen receptor activation during prostate cancer progression. Oncogene.

[7] Xie D, Gore C, Liu J (2010). Role of DAB2IP in modulating epithelial-to-mesenchymal transition and prostate cancer metastasis. Proc Natl Acad Sci USA.

[8] Min J, Zaslavsky A, Fedele G (2010). An oncogene-tumor suppressor cascade drives metastatic prostate cancer by coordinately activating Ras and nuclear factor-kappaB. Nat Med.

[9] Wang Z, Tseng CP, Pong RC (2002). The mechanism of growth-inhibitory effect of DOC-2/DAB2 in prostate cancer. Characterization of a novel GTPase-activating protein associated with N-terminal domain of DOC-2/DAB2. J Biol Chem.

[10] Wu K, Xie D, Zou Y (2013). The mechanism of DAB2IP in chemoresistance of prostate cancer cells. Clin Cancer Res.

[11] Kong Z, Raghavan P, Xie D (2010). Epothilone B confers radiation dose enhancement in DAB2IP gene knock-down radioresistant prostate cancer cells. Int J Radiat Oncol Biol Phys.

[12] Yu L, Tumati V, Tseng SF (2012). DAB2IP regulates autophagy in prostate cancer in response to combined treatment of radiation and a DNA-PKcs inhibitor. Neoplasia (New York, NY).

[13] Bartel DP (2004). MicroRNAs: Genomics, biogenesis, mechanism and function. Cell.

[14] Calin GA, Croce CM (2006). MicroRNA signatures in human cancers. Nat Rev Cancer.

[15] Tutar L, Tutar E, Tutar Y (2014). MicroRNAs and cancer; an overview. Curr Pharm Biotechnol.

[16] Walter BA, Valera VA, Pinto PA, Merino MJ (2013). Comprehensive microRNA profiling of prostate cancer. J Cancer.

[17] Leung CM, Li SC, Chen TW (2014). Comprehensive microRNA profiling of prostate cancer cells after ionizing radiation treatment. Oncol Rep.

[18] Liu K, Huang J, Xie M (2014). MIR34A regulates autophagy and apoptosis by targeting HMGB1 in the retinoblastoma cell. Autophagy.

[19] Liu B, Che W, Xue J (2013). SIRT4 prevents hypoxia-induced apoptosis in H9c2 cardiomyoblast cells. Cell Physiol Biochem.

[20] Tanida I, Ueno T, Kominami E (2004). LC3 conjugation system in mammalian autophagy. Int J Biochem Cell Biol.

[21] Sou YS, Tanida I, Komatsu M, Ueno T, Kominami E (2006). Phosphatidylserine in addition to phosphatidylethanolamine is an in vitro target of the mammalian Atg8 modifiers, LC3, GABARAP and GATE-16. J Biol Chem.

[22] Rouschop KM, van den Beucken T, Dubois L (2010). The unfolded protein response protects human tumor cells during hypoxia through regulation of the autophagy genes MAP1LC3B and ATG5. J Clin Invest.

[23] Kondo Y, Kanzawa T, Sawaya R, Kondo S (2005). The role of autophagy in cancer development and response to therapy. Nat Rev Cancer.

[24] Jung CH, Kim H, Ahn J, Jung SK, Um MY, Son KH, Kim TW, Ha TY (2013). Anthricin isolated from *Anthriscus sylvestris (L.) Hoffm. Inhibits the growth of breast cancer cells by inhibiting Akt/mTOR signaling and its apoptotic effects are enhanced by autophagy inhibition*. Evid Based Complement Alternat Med.

[25] Zoncu R, Efeyan A, Sabatini DM (2011). mTOR: From growth signal integration to cancer, diabetes and ageing. Nat Rev Mol Cell Biol.

[26] Zeng X, Kinsella TJ (2008). Mammalian target of rapamycin and S6 kinase 1 positively regulate 6-thioguanine-induced autophagy. Cancer Res.

[27] Scott RC, Schuldiner O, Neufeld TP (2004). Role and regulation of starvation-induced autophagy in the Drosophila fat body. Dev Cell.

[28] Klionsky DJ, Meijer AJ, Codogno P (2005). Autophagy and p70S6 kinase. Autophagy.

[29] Ambs S, Prueitt RL, Yi M, Hudson RS, Howe TM, Petrocca F, Wallace TA, Liu CG, Volinia S, Calin GA (2008). Genomic profiling of microRNA and messenger RNA reveals deregulated microRNA expression in prostate cancer. Cancer Res.

[30] Paller CJ, Antonarakis ES, Eisenberger MA, Carducci MA (2013). Management of patients with biochemical recurrence after local therapy for prostate cancer. Hematol Oncol Clin North Am.

[31] Orth M, Lauber K, Niyazi M, Friedl AA, Li M, Maihöfer C, Schüttrumpf L, Ernst A, Niemöller OM, Belka C (2014). Current concepts in clinical radiation oncology. Radiat Environ Biophys.

[32] Kaliberov SA, Buchsbaum DJ (2012). Chapter seven - Cancer treatment with gene therapy and radiation therapy. Adv Cancer Res.

[33] Huber TB, Edelstein CL, Hartleben B, Inoki K, Jiang M, Koya D, Kume S, Lieberthal W, Pallet N, Quiroga A (2012). Emerging role of autophagy in kidney function, diseases and aging. Autophagy.

[34] Hale AN, Ledbetter DJ, Gawriluk TR, Rucker EB (2013). 3rd: Autophagy: Regulation and role in development. Autophagy.

[35] Massoner P, Thomm T, Mack B, Untergasser G, Martowicz A, Bobowski K, Klocker H, Gires O, Puhr M (2014). EpCAM is overexpressed in local and metastatic prostate cancer, suppressed by chemotherapy and modulated by MET-associated miRNA-200c/205. Br J Cancer.

[36] Schmukler E, Shai B, Ehrlich M, Pinkas-Kramarski R (2012). Neuregulin promotes incomplete autophagy of prostate cancer cells that is independent of mTOR pathway inhibition. PLoS One.

[37] Livesey KM, Tang D, Zeh HJ, Lotze MT (2009). Autophagy inhibition in combination cancer treatment. Curr Opin Investig Drugs.

[38] Chen H, Tu SW, Hsieh JT (2005). Down-regulation of human DAB2IP gene expression mediated by polycomb Ezh2 complex and histone deacetylase in prostate cancer. J Biol Chem.

[39] Nishikawa R, Goto Y, Sakamoto S, Chiyomaru T, Enokida H, Kojima S, Kinoshita T, Yamamoto N, Nakagawa M, Naya Y (2014). Tumor-suppressive microRNA-218 inhibits cancer cell migration and invasion via targeting of LASP1 in prostate cancer. Cancer Sci.

[40] Qiang XF, Zhang ZW, Liu Q, Sun N, Pan LL, Shen J, Li T, Yun C, Li H, Shi LH (2014). miR-20a promotes prostate cancer invasion and migration through targeting ABL2. J Cell Biochem.

[41] Shen PF, Chen XQ, Liao YC, Chen N, Zhou Q, Wei Q, Li X, Wang J, Zeng H (2014). MicroRNA-494-3p targets CXCR4 to suppress the proliferation, invasion and migration of prostate cancer. Prostate.

[42] Kang S, Zhao Y, Hu K, Xu C, Wang L, Liu J, Yao A, Zhang H, Cao F (2014). miR-124 exhibits antiproliferative and antiaggressive effects on prostate cancer cells through PACE4 pathway. Prostate.

[43] Surviladze Z, Sterk RT, DeHaro SA, Ozbun MA (2013). Cellular entry of human papillomavirus type 16 involves activation of the phosphatidylinositol 3-kinase/Akt/mTOR pathway and inhibition of autophagy. J Virol.

